# Complete nondestructive analysis of two-photon six-qubit hyperentangled Bell states assisted by cross-Kerr nonlinearity

**DOI:** 10.1038/srep22016

**Published:** 2016-02-25

**Authors:** Qian Liu, Guan-Yu Wang, Qing Ai, Mei Zhang, Fu-Guo Deng

**Affiliations:** 1 Department of Physics, Applied Optics Beijing Area Major Laboratory, Beijing Normal University, Beijing 100875, China

## Abstract

Hyperentanglement, the entanglement in several degrees of freedom (DOFs) of a quantum system, has attracted much attention as it can be used to increase both the channel capacity of quantum communication and its security largely. Here, we present the first scheme to completely distinguish the hyperentangled Bell states of two-photon systems in three DOFs with the help of cross-Kerr nonlinearity without destruction, including two longitudinal momentum DOFs and the polarization DOF. We use cross-Kerr nonlinearity to construct quantum nondemolition detectors which can be used to make a parity-check measurement and analyze Bell states of two-photon systems in different DOFs. Our complete scheme for two-photon six-qubit hyperentangled Bell-state analysis may be useful for the practical applications in quantum information, especially in long-distance high-capacity quantum communication.

Quantum entanglement plays an important role in quantum information processing. It is the key resource for quantum communication tasks, such as quantum teleportation^1^, quantum swapping^2^, quantum dense coding^3^,^4^, quantum key distribution^5^,^6^, quantum secret sharing^7^, quantum secure direct communication^8^,^9^,^10^,^11^,^12^, and so on. Recently, hyperentanglement, the entanglement in multiple degrees of freedom of a quantum system^13^,^14^,^15^, has attracted much attention. It can be used to complete the deterministic entanglement purification for nonlocal photonic systems in the polarization degree of freedom (DOF)^16^,^17^,^18^,^19^,^20^, which reduces largely the resource consumed for quantum repeaters. As it is impossible to deterministically distinguish the four Bell states in polarization with only linear optical elements, hyperentanglement can also used to assist the complete Bell-state analysis (BSA)^16^,^17^. For instance, Kwiat and Weinfurter^21^ proposed a BSA scheme using photons entangled in polarization and momentum (spatial mode) in 1998. In 2003, Walborn *et al.*^22^ presented a simple linear-optical scheme for the complete Bell-state analysis of photons with hyperentanglement in both polarization and momentum. The experiments of a complete BSA with polarization-time-bin hyperentanglement^23^ and polarization-momentum hyperentanglement^24^ have also been reported in succession. For all the linear-optical BSA protocols mentioned above, they use one DOF as an ancillary to accomplish the complete BSA in the other DOF, rather than distinguish all the hyperentangled Bell states themselves. In 2007, Wei *et al.*^25^ pointed out that 7 states in the group of 16 orthogonal hyperentangled Bell states can be distinguished with only linear optics. The general theoretical explanation has been presented by Pisenti’s group^26^ in 2011.

Hyperentanglement of photon systems can increase both the channel capacity of long-distance quantum communication and its security. In 2008, Barreiro *et al.*^27^ beat the channel capacity limit for linear photonic superdense coding with polarization-orbital-angular-momentum hyperentanglement. In 2012, Wang, Song, and Long^28^ proposed an efficient quantum repeater protocol for long-distance quantum communication with hyperentanglement. In 2013, Ren, Du, and Deng^29^ gave the first hyperentanglement concentration protocol (hyper-ECP) for two-photon four-qubit systems with linear optics. In the same year, Ren and Deng^30^ proposed the original hyperentanglement purification protocol (HEPP) for polarization-spatial hyperentangled states assisted by diamond nitrogen-vacancy centers inside photonic crystal cavities. In 2014, Ren, Du, and Deng^31^ gave a two-step HEPP for polarization-spatial hyperentangled states with the quantum-state-joining method, and it has a far higher efficiency. Ren and Long^32^ proposed a general hyper-ECP for photon systems assisted by quantum dot spins inside optical microcavities. Li and Ghose^33^ presented a hyper-ECP for multipartite entanglement via linear optics. Some other interesting protocols for hyperentanglement concentration and hyperentanglement purification^34^,^35^,^36^ were presented in 2015.

In fact, in long-distance high-capacity quantum communication, the complete analysis for all the orthogonal hyperentangled Bell states of photon systems in multiple DOFs is necessary. The 16 orthogonal hyperentangled Bell states of two-photon systems in two DOFs can be distinguished completely if nonlinear optics is introduced. In 2010, Sheng *et al.*^37^ gave the first scheme for the complete hyperentangled-Bell-state analysis (HBSA) for quantum communication with the help of cross-Kerr nonlinearity. In 2012, Ren *et al.*^38^ proposed another complete HBSA scheme for photon systems in both the polarization and the spatial-mode DOFs with the help of giant nonlinear optics in one-sided quantum-dot-cavity systems. Using double-sided quantum-dot-cavity systems, the complete HBSA scheme also can be accomplished^39^. Xia *et al.*^40^ proposed an efficient scheme for hyperentangled Greenberger-Horne-Zeilinger-state analysis with cross-Kerr nonlinearity. Recently, the hyperentangled Bell states for two-photon six-qubit systems were produced in experiments^41^,^42^, but there are no schemes for the complete analysis on two-photon six-qubit quantum states as they are far more difficult, compared with the Bell states in both one and two DOFs.

In this paper, we give the first scheme to completely distinguish the hyperentangled Bell states of two-photon systems in three DOFs with the help of cross-Kerr nonlinearity without destruction, including a polarization DOF and double longitudinal momentum DOFs. Our HBSA protocol for two-photon six-qubit hyperentangled systems may be useful in the practical applications in quantum information processing, blind quantum computation, distributed quantum computation, and especially long-distance high-capacity quantum communication in the future. With hyperdense coding on two-photon systems entangled in three DOFs simultaneously as an example, we show the principle of the applications of our HBSA protocol in detail.

## Results

### Complete analysis for the states of a two-photon system in momentum modes

A hyperentangled Bell state of two-photon six-qubit systems in three DOFs can be described as follows:


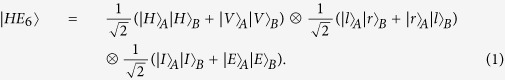


**Figure 1:**
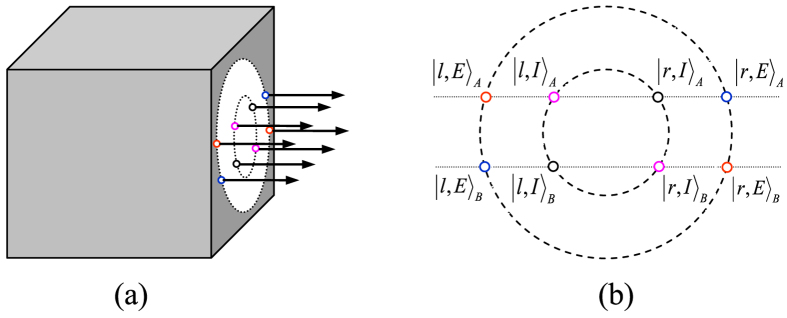
(**a**) Source for two-photon six-qubit hyperentangled Bell states. A detailed description of the source is given in the previous work^42^. (**b**) Modes for two-photon six-qubit hyperentangled Bell states. The upper modes correspond to Alice’s photon, while the lower modes correspond to Bob’s photon. *l*, *r*, *I* and *E* are the left, right, internal, and external modes for a photon, respectively.

Here the subscripts A and B denote the two photons. *H* and *V* represent the horizontal and the vertical polarizations of photons, respectively. The three independent DOFs are polarization and a double longitudinal momentum (*r*/*l* and *E*/*I*), shown in Fig. 1. The system of the two-photon six-qubit source^42^ consists of two type-1 *β* barium borate (BBO) crystal slabs and a eight-hole screen. When a continuous-wave (cw) vertically polarized *Ar*^+^ laser beam interacts through spontaneous parametric down-conversation (SPDC) with the two BBO crystal slabs, and the nonlinear interaction between the laser beam and the BBO crystal leads to the production of the degenerate photon pairs, which are entangled in polarization and belong to the surfaces of two emission cones. As shown in Fig. 1(a), the insertion of a eight-hole screen allows us to achieve the double longitudinal momentum entanglement. The labels in Fig. 1(b) are used to identify the selected modes. The internal (*I*) and the external (*E*) cones correspond to the first and the second crystals, respectively. Furthermore, *l* (*r*) refers to the left (right) side of each cone.

The distinction between the internal (*I*) and the external (*E*) modes provides us the second longitudinal momentum DOF, while the first longitudinal momentum DOF comes from the distinction between the left (*l*) and right (*r*) modes. Therefore, the six-qubit hyperentangled state described in Eq. (1) is given by the product of one polarization entangled state and two longitudinal momentum entangled states of a photon pair.

Let us denote the four Bell states in the polarization DOF of two-photon systems as


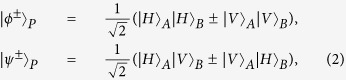


and four Bell states in the first longitudinal momentum DOF as


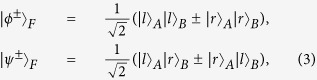


while the four Bell states in the second longitudinal momentum DOF can be expressed as


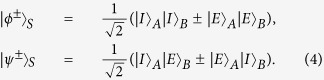


Here the subscripts *P*, *F*, and *S* denote the polarization, the first longitudinal momentum, and the second longitudinal momentum DOFs of a two-photon six-qubit system, respectively.

**Figure 2:**
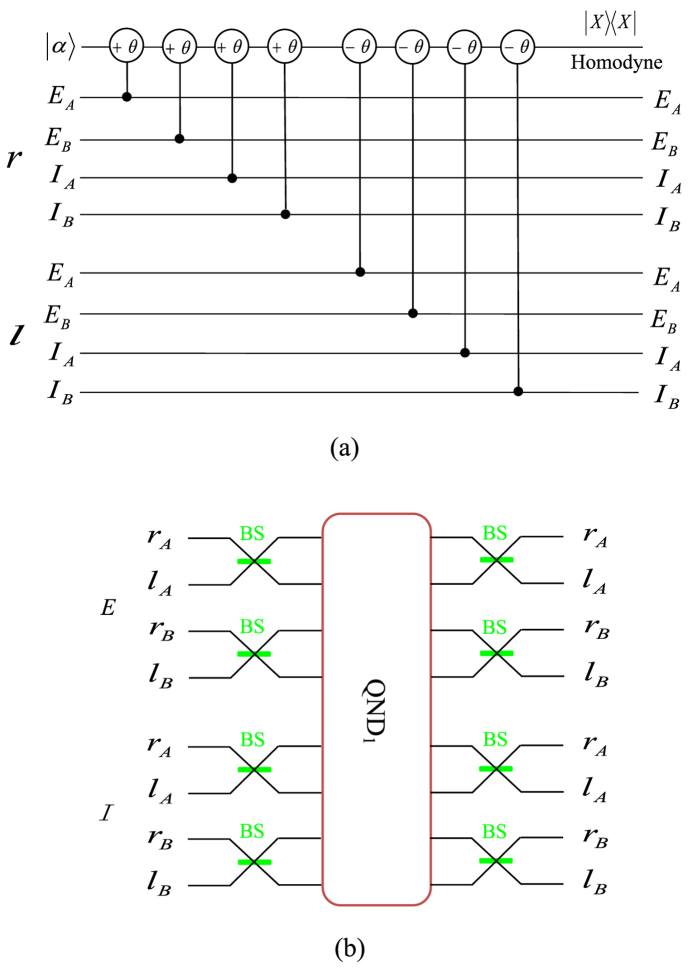
(**a**) Schematic diagram of QND_1_ which is used to distinguish the even-parity states 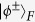 from the odd-parity states 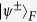 in the first longitudinal momentum DOF of the two-photon six-qubit system *AB*. ±*θ* denotes the cross-Kerr nonlinear media which will make the cohere probe beam 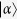 have a phase shift ±*θ* when there is a signal photon passing through it. 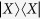 is the homodyne measurement to discriminate different phase shifts of the coherent probe beam. *r* and *l* represent the left and the right sides of each cone from where the photons emit, respectively. The internal (*I*) and the external (*E*) cones correspond to the first and the second crystal from which the photons are produced, respectively. (**b**) Schematic diagram of QND_2_. Each of the 50:50 BSs acts as a Hadamard operation 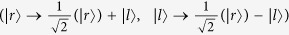 on the photon in the first longitudinal momentum DOF. After these two photons pass through the BSs, one can use mirrors to separate the paths of photons.

The principle of our scheme for the complete analysis on the quantum states of a two-photon six-qubit system in the first longitudinal momentum DOF is shown in Fig. 2. In detail, one can let the two photons *AB* pass through the first quantum nondemolition detector (QND_1_) whose circuit is shown in Fig. 2(a). Based on the principle of cross-Kerr effect (see Methods), the evolution of two-photon six-qubit hyperentangled Bell states and the coherent state can be described as follows:


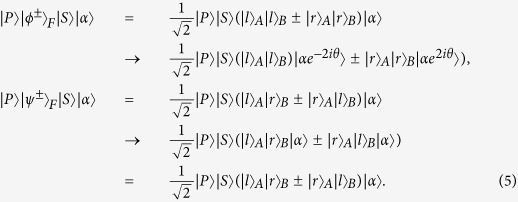


Here, 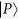 represents the four Bell stats in the polariztion DOF, and 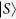 denotes the four Bell states in the second longitudinal momentum DOF. The equation above shows that the Bell states of other two DOFs have not changed. If these two photons are in the same state 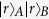 or 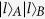 in the first longitudinal momentum DOF, the coherent probe beam will pick up a phase shift +2*θ* or −2*θ*. If these two photons are in the different states 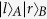 or 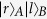, the phase shift of the coherent probe beam will be 0. As the homodyne measurement cannot distinguish +2*θ* from −2*θ*, there are only two measurement outcomes 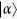 and 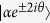 for the coherent probe beam. Thus, according to the measurement results, one can distinguish the even-parity states 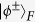 from the odd-parity states 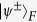. That is, QND_1_ shown in Fig. 2(a) is a quantum nondemolition detector, with which one can distinguish the parity of the two photons *A* and *B* in the first longitudinal momentum DOF.

After QND_1_, one can divide the four Bell states in the first longitudinal momentum DOF into two groups, 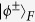 and 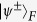. The next task is to distinguish the different phases in 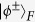 and 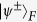, respectively. By using the 50:50 beam splitters (BSs) shown in Fig. 2(b) on the photons, one can get the following transformations:


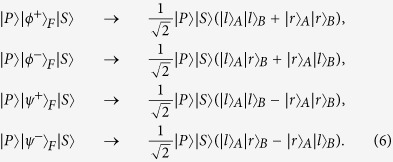


As the BSs transform the phase difference of the two states from each group into the parity difference, the two Bell states in the same group will belong to different groups after the BSs. Then, if we let photon A and photon B pass through the same quantum circuit as QND_1_ shown in Fig. 2(b), the four Bell states can be distinguished completely. Although the states 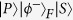 and 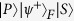 have changed into 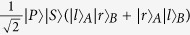 and 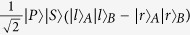 by BSs in this procedure, respectively, one can use other BSs after the quantum circuit as QND_1_ to recover the initial Bell states in the first longitudinal momentum DOF. The relationship between the measurement results of these two QNDs and the corresponding Bell states in the first longitudinal momentum DOF is shown in Table 1.

**Figure 3:**
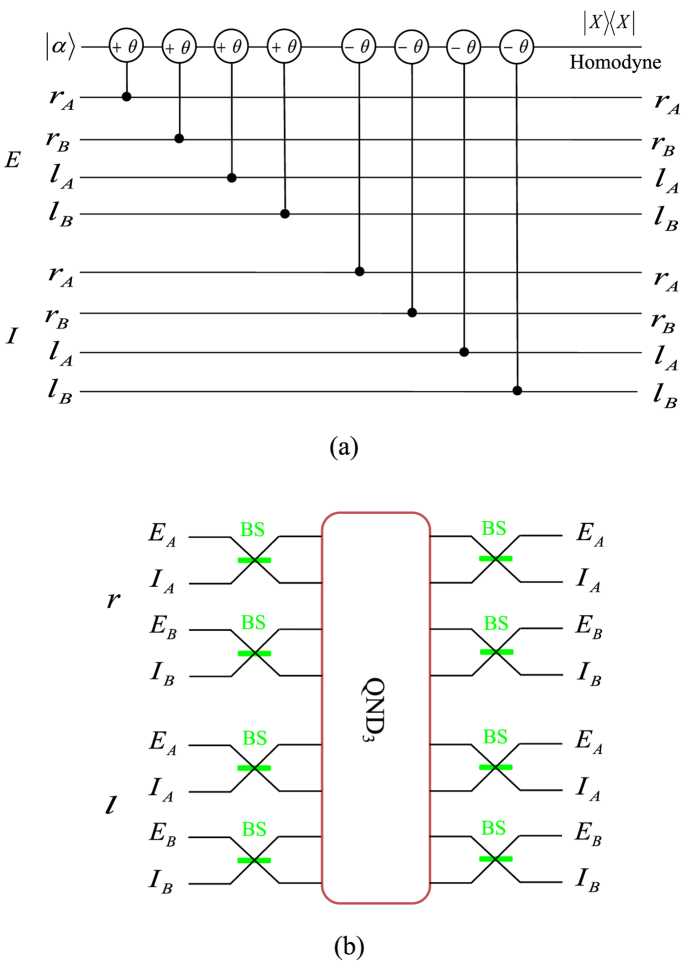
Schematic diagram for distinguishing the four Bell states of the two-photon six-qubit system in the second longitudinal momentum DOF. (**a**) QND_3_. (**b**) QND_4_, the 50:50 BS is used to perform the Hadamard operation 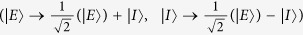 on the second longitudinal momentum DOF of photons.

Now, we have finished the distinction of the four Bell states in the first longitudinal momentum, without destroying the hyperentanglement in the other two DOFs. Then we move to the next step to distinguish the four Bell states in the second longitudinal momentum DOF. As the first longitudinal momentum and the second longitudinal momentum are all linear momentum, what we do to realize the next distinction is similar to the analysis protocol of the first longitudinal momentum DOF. The difference is to interchange the path labels *r*/*l* to *E*/*I*. The principle for distinguishing the four Bell states of the two-photon system in the second longitudinal momentum DOF is shown in Fig. 3. Here, we let the two photons pass through QND_3_ and then QND_4_ in sequence. With these two QNDs, we can analyze the four Bell states in the second longitudinal momentum DOF completely. The relationship between the measurement results of this scheme and the corresponding Bell states in the second longitudinal momentum DOF is described in Table 2.

### Complete six-qubit hyperentangled Bell state analysis scheme for states in polarization

**Figure 4:**
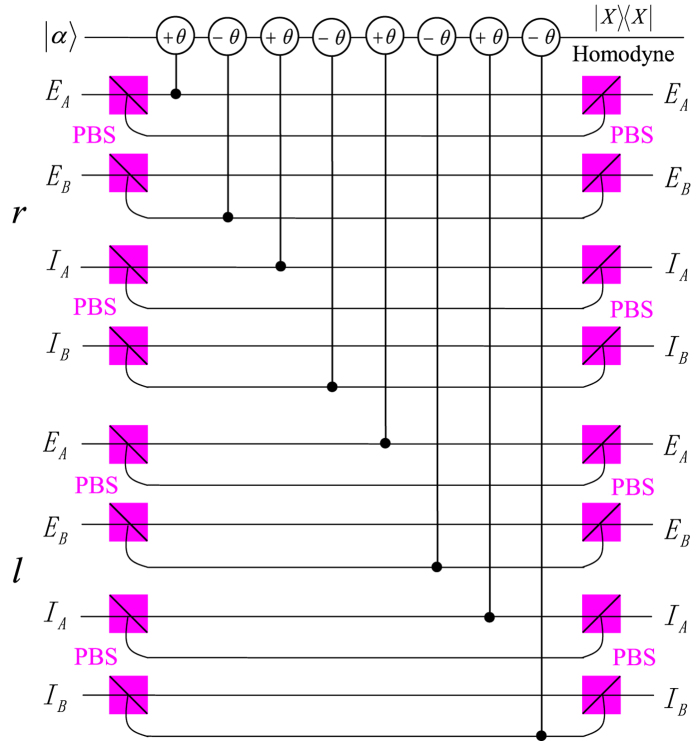
Schematic diagram of QND_5_ which is used to distinguish the even-parity states 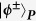 from the odd-parity states 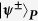 in polarization DOF of the two-photon six-qubit system *AB*. PBS represents a polarizing beam splitter which is used to transmit the horizontal (*H*) polarization photon and reflect the vertical (*V*) polarization photon, respectively.

**Figure 5:**
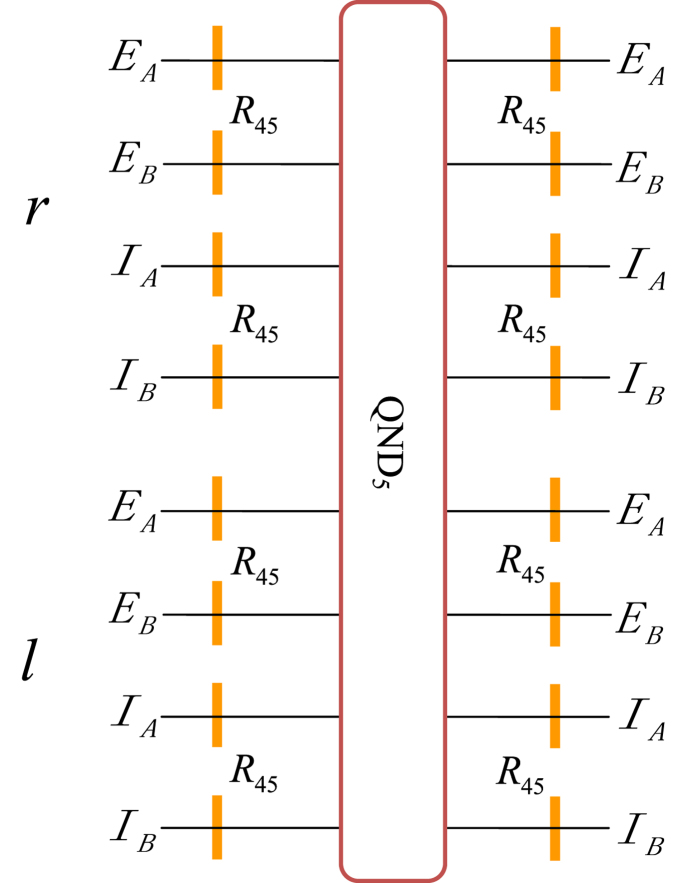
Schematic diagram of QND_6_. *R*_45_ represents the wave plate which rotates the horizontal and vertical polarizations by 45° to accomplish a Hadamard operation 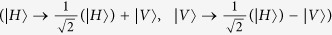 on polarization of photons.

Now, let us move our attention to the last task, which is to distinguish the four Bell states of the two-photon six-qubit system in the polarization DOF. The analysis of the four Bell states in polarization is analogous to that in previous works^37^,^43^. The schematic diagram for the distinction of the four Bell states in polarization is shown in Figs 4 and 5.

According to QND_5_ shown in Fig. 4, the states 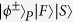 with the coherent state 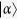 evolve as


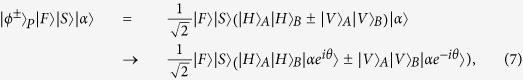


while the states 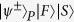 with the coherent state 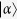 evolve as


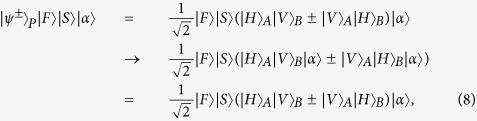


where 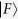 represents the four Bell states in the first longitudinal momentum DOF. In these evolutions, the modes 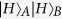 or 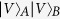 will let the coherent probe beam pick up a phase shift +*θ* or −*θ*, but the coherent probe beam will pick up no phase shift if the two photons are in the mode 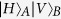 or 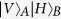. With an X-quadrature measurement on the coherent beam, as 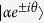 cannot be distinguished, one can divide the four Bell states in polarization into two groups, the even-parity one 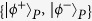 and the odd-parity one 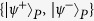.

The next step is to distinguish the different relative phases in each of these two groups. This task can be accomplished with the circuit shown in Fig. 5. Here the wave plate *R*_45_ is used to accomplish a Hadamard operation on the polarization of photons. A Hadamard operation on each of the two photons *AB* will make the following transformations:


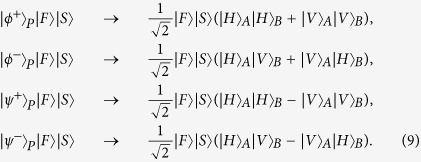


As *R*_45_ can transform the phase difference into the parity difference, one can then use the same quantum circuit as QND_5_ to distinguish the parity difference between the two states in each group. Then we use other *R*_45_ to recover the initial Bell states in polarization DOF. That is, after the photons pass through QND_6_ shown in Fig. 5, the two Bell states in the even-parity group 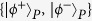 or the odd-parity one 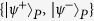 can be distinguished completely. The relationship between the measurement results of this scheme and the corresponding Bell states in polarization is described in Table 3.

From the analysis above, one can see that the complete nondestructive analysis for two-photon six-qubit hyperentangled Bell states can be accomplished with the sequential connection of the six QNDs. This complete HBSA can be used to complete some other important tasks in high-capacity quantum communication, such as teleportation with photon systems in three DOFs, hyperentanglement swapping, quantum hyperdense coding, and so on.

## Discussion

In our six-qubit HBSA scheme, we exploit the cross-Kerr nonlinearity to construct the QNDs to check the parity of the two photons in the three DOFs. Therefore, we should acknowledge that the feasibility of the proposed scheme depends on the nonlinear phase shift of the Kerr media. Although many works have been reported on cross-Kerr nonlinearity^44^,^45^,^46^, a clean cross-Kerr nonlinearity in the optical single-photon regime is quite a controversial assumption with current technology. In 2006 and 2007, Shapiro and Razavr^47^,^48^ pointed out that the single-photon Kerr nonlinearity may do no help in quantum computation. Moreover, in 2010, the research results of Gea-Banacloche^49^ suggested that a large phase shift via a “giant Kerr effect” with single-photon wave packets is impossible at present.

Fortunately, our HBSA scheme only requires a small phase shift, as long as it can be distinguished from zero, and much progress has been made on the Kerr nonlinearity and homodyne detection. In 2003, Hofmann *et al.*^50^ demonstrated that a phase shift of *π* can be achieved with a single two-level atom one-sided cavity system. In 2010, Wittmann *et al.*^51^ investigated the difference between a standard homodyne detector and a displacement-controlled photon number resolving (PNR) detector. They showed that the displacement-based PNR receiver outperforms the standard homodyne detection. Therefore, for a weak cross-Kerr nonlinearity 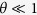, if we choose a sufficiently large amplitude of the coherent state, which satisfies the requirement 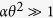, it is possible for us to achieve deterministic distinguishability between the shifted and non-shifted phases in the coherent state. Furthermore, in 2011, He *et al.*^46^ showed that effects due to the transverse degrees of freedom significantly affect the cross-phase modulation process, and made the treatment of single-photon-coherent-state interactions more realistic. In the same year, Feizpour *et al.*^52^ researched the cross-Kerr nonlinearity between continuous-mode coherent states and single photons, and they indicated that a cross-Kerr phase shift is likely to be amplified to observable value with weak-value amplification. Moreover, Zhu and Huang^53^ showed that giant Kerr nonlinearity of the probe and the signal pulses may be achieved with nearly vanishing optical absorption. The substantial cross-Kerr nonlinearities^54^,^55^ have already been obtained in the microwave domain using superconducting qubits. In the work by Hoi *et al.*^54^, the average cross-Kerr phase shift was demonstrated up to 20 degrees per photon with both coherent microwave fields at the single-photon level.

Before ending this work, we will briefly discuss the application of our HBSA scheme in quantum hyperdense coding. As quantum hyperdense coding is the generalization of quantum dense coding with photon systems in several DOFs, with our six-qubit HBSA scheme, one can transfer six bits of classical information by sending only one photon. In order to realize quantum hyperdense coding, the sender must choose one of the local 64 operations 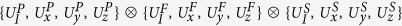 to perform on photon, in which 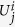 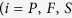 and 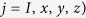 are unitary operations in polarization or one of the two longitudinal momentum DOFs. Here, the unitary operations 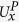 and 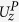 can be achieved by a half-wave plate set at 45° and 0°, respectively. The combination of 0° and 45° half-wave plates can be used to perform the unitary operation 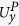. One can accomplish the operation 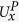, 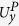, and 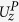 by putting appropriate half-wave plates in all the four paths of the photon. 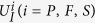 is unit operation, which means doing nothing on the photon. For single-photon longitudinal-momentum states, one can exchange the two modes to accomplish the operation 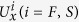. The operation 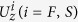 can be achieved by putting 0° half-wave plates in the appropriate path. The operation 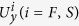 is the combination of 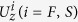 and 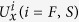. Using those operations and our six-qubit HBSA scheme, we can accomplish the six-bit quantum hyperdense coding which will largely improve the capacity of long-distance quantum communication.

In summary, we have proposed an efficient scheme for the complete nondestructive analysis of hyperentanglement of two-photon systems in three DOFs with the help of the cross-Kerr nonlinearity. We use cross-Kerr nonlinearity to construct quantum nondemolition detectors which are used to make a parity-check measurement and analyze Bell states in different DOFs of two-photon systems. We have also presented the applications of our HBSA protocol in quantum hyperdense coding with two-photon systems entangled in three DOFs simultaneously, which means that our HBSA protocol may be useful for practical applications in quantum information processing, blind quantum computation, distributed quantum computation, and especially long-distance high-capacity quantum communication in future.

## Methods

### Cross-Kerr nonlinearity

The Hamiltonian of a cross-Kerr nonlinearity medium is^44^,^56^


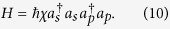


Here 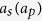 and 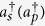 are the annihilation and the creation operators of the signal (probe) pulse beam, respectively. 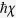 is the coupling strength of the nonlinearity, which is decided by the property of the nonlinear material. If we consider that the probe beam is the coherent state 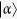, for an arbitrary signal state 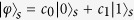, the effect of the cross-Kerr nonlinearity on the whole system can be described as


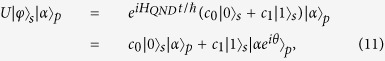


where 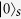 and 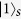 are the Fock states for the signal pulse. The phase shift 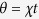 and *t* is the interaction time which is proportional to the number of photons with the single-photon state being unaffected.

## Additional Information

**How to cite this article**: Liu, Q. *et al.* Complete nondestructive analysis of two-photon six-qubit hyperentangled Bell states assisted by cross-Kerr nonlinearity. *Sci. Rep.* **6**, 22016; doi: 10.1038/srep22016 (2016).

## Figures and Tables

**Table 1:** The relationship between the four Bell states in the first longitudinal momentum DOF and the measurement results of QND_1_ and QND_2_.

Bell states	QND_1_	QND_2_
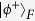	±2*θ*	±2*θ*
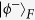	±2*θ*	0
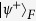	0	±2*θ*
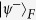	0	0

**Table 2:** The relationship between the four Bell states in the second longitudinal momentum DOF and the measurement results of QND_3_ and QND_4_.

Bell states	QND_3_	QND_4_
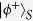	±2*θ*	±2*θ*
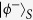	±2*θ*	0
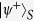	0	±2*θ*
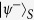	0	0

**Table 3:** The relationship between the four Bell states in the polarization DOF and the measurement results of QND_5_ and QND_6_.

Bell states	QND_5_	QND_6_
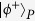	±*θ*	±*θ*
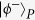	±*θ*	0
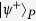	0	±*θ*
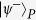	0	0
